# A comprehensive full-length transcriptome landscape of cigarette smoke-exposed HASMCs reveals extensive remodeling of mRNA isoforms and regulatory networks

**DOI:** 10.1371/journal.pone.0354604

**Published:** 2026-09-21

**Authors:** Wei Gou, Lei Yang, Shan Chen, Yulin Miao, Weiwei Wang, Hai Li, Zhipeng Hu

**Affiliations:** 1 Department of Vascular Surgery, General Hospital of Ningxia Medical University, Yinchuan City, Ningxia, China; 2 Department of Colorectal Surgery, General Hospital of Ningxia Medical University, Yinchuan City, Ningxia, China; UC Los Angeles: University of California Los Angeles, UNITED STATES OF AMERICA

## Abstract

Cigarette smoke extract (CSE) exerts paradoxical concentration-dependent effects on vascular smooth muscle cells, though the underlying isoform-level mechanisms remain unexplored. We employed Oxford Nanopore long-read sequencing to analyze the full-length transcriptome of human aortic smooth muscle cells after CSE treatment. We confirmed that low-concentration CSE (0.2%) slightly promoted cell viability while higher concentrations (0.4%−0.8%) induced dose-dependent suppression. Sequencing of 0.6% CSE (midpoint cytotoxicity)-treated cells revealed 11,222 novel transcripts and extensive post-transcriptional reprogramming, including: 1,866 upregulated transcripts involving ferroptosis and cell division; 493 downregulated transcripts in oxidative phosphorylation and glutathione metabolism; 1,753 alternative polyadenylation events mediating 3’UTR remodeling; and 269 alternative splicing events affecting cell cycle regulators and transcription factors (CREM, FOSL1, HBP1). Our study suggests that high-concentration CSE cytotoxicity is executed through coordinated post-transcriptional dysregulation, providing the first isoform-level perspective on smoking-induced vascular pathology and revealing previously unrecognized regulatory mechanisms in cigarette smoke-mediated vascular injury.

## 1. Introduction

Cigarette smoking constitutes a paramount global health challenge and a leading risk factor for cardiovascular, respiratory, and neoplastic diseases, accounting for an immense burden of morbidity and mortality worldwide [1–5]. The pathophysiological consequences of smoking have been extensively documented, with its cessation demonstrably reversing many adverse effects and significantly reducing associated mortality [2,6]. At the molecular level, transcriptomic studies have revealed that while the expression of most smoking-deregulated genes normalizes upon cessation, a subset of genes persists in an altered state, suggesting long-lasting molecular scars [7,8]. However, the prevailing focus of these investigations has been on alterations in gene-level expression, leaving a critical gap in our understanding of smoking’s impact on the regulatory layer of transcript isoform diversity.

The vast majority of multi-exon human genes undergo alternative isoform regulation, primarily through mechanisms such as alternative splicing (AS) and alternative polyadenylation (APA). These processes profoundly expand the functional complexity of the genome by generating distinct transcript variants from a single gene, which can exhibit differences in stability, localization, and protein-coding potential, thereby playing pivotal roles in cell identity and disease pathogenesis [9–13]. Emerging evidence from in vivo studies hints at the significance of this regulatory axis; for instance, an RNA-seq analysis of human smokers’ blood cell mRNA/lncRNA identified differential exon usage, particularly in the first and last exons, suggesting widespread alterations in transcription initiation and termination [14]. A subsequent larger study further confirmed a pervasive transcriptomic switch in smokers towards isoforms with longer 3’ untranslated regions (3’UTRs), mediated by APA, which consequently introduced additional regulatory elements like microRNA (miRNA) and RNA-binding proteins binding sites [15]. However, the mRNA/lncRNA isoform information from these previous studies on human blood samples was based on the computational analysis of the short-read sequencing data, which lack full-length isoform information. Moreover, despite these compelling observations in human cohorts, the direct causal impact of cigarette smoke constituents on isoform regulation in specific cell types central to smoking-related diseases remains largely unexplored.

Vascular smooth muscle cells (VSMCs) are crucial mediators of vascular homeostasis and key players in the pathogenesis of smoking-induced cardiovascular diseases, such as atherosclerosis and abdominal aortic aneurysm. Crucially, VSMCs are not terminally differentiated and can undergo phenotypic switching from a contractile to a synthetic, proliferative, and migratory state in response to pathological stimuli—a process in which post-transcriptional regulation is critically involved [16–18]. Therefore, elucidating how cigarette smoke directly reprograms the VSMC transcriptome at the full-length isoform level is critical for understanding the molecular underpinnings of vascular pathology.

To address this, we employed an in vitro model treating human aortic smooth muscle cells (HASMCs) with cigarette smoke extract (CSE) to directly simulate the exposure. Several studies have indicated that CSE promotes VSMC proliferation, while Sampilvanjil et al. have shown it induces ferroptosis [19–22]. However, we found that higher concentrations of CSE significantly suppressed HASMC proliferation, exhibiting a clear dose-dependent relationship. Moving beyond traditional short-read sequencing, we utilized Oxford Nanopore Technologies (ONT) long-read sequencing, which provides full-length transcript information and is uniquely powerful for the comprehensive and unambiguous detection of transcript isoforms, AS events, APA sites, and 3’UTR lengths. Our study yielded a high-resolution map of the CSE-induced alterations in the HASMC transcriptome. We report the discovery of a vast repertoire of differential transcript isoforms, widespread changes in AS and APA patterns, and the identification of numerous novel transcripts. These findings indicate the direct and substantial impact of CSE on the post-transcriptional regulatory landscape of HASMCs, unveiling a previously underappreciated mechanism through which smoking may drive vascular dysfunction and disease.

## 2. Materials and methods

### 2.1. Preparation of cigarette smoke extract

In this study, Huanghelou brand was selected as a representative commercially available cigarette with tar and nicotine content (10 mg and 0.9 mg per cigarette, respectively) comparable to standard research cigarettes used in previous studies [19–21]. We acknowledge that different cigarette brands may have compositional variations, which represents a limitation of this study. Cigarette smoke extract (CSE) was prepared using a negative-pressure suction apparatus, following previously reported methods [19]. Ten cigarettes were continuously combusted, and the generated smoke was drawn into a sealed flask containing 10 mL of serum-free DMEM using a 50 mL syringe under negative pressure. Each cigarette was allowed to burn for approximately 2 minutes. Smoke collection was stopped after all ten cigarettes had been exhausted through the medium. The resulting solution was adjusted to a pH of 7.3–7.4 with sodium hydroxide (NaOH), passed through a 0.22 μm filter to remove bacteria and particulate matter, and its absorbance at 340 nm was measured using a microplate reader (FC, Thermo). An absorbance value of approximately 2.6 was defined as 100% CSE. The CSE stock solution was aliquoted and stored at −80 °C until use. Working concentrations of CSE were freshly prepared by diluting the stock solution with serum-free medium and were used within 30 minutes of preparation.

### 2.2. CSE treatment of human aortic smooth muscle cells (HASMCs)

The immortalized human aortic smooth muscle cell line (HASMC, CP-H081Y) was purchased from Wuhan Procell Biotechnology Co., Ltd. The cell line was authenticated by the supplier and tested negative for mycoplasma contamination. As this study used a commercially available cell line with no human or animal subjects, ethical approval was not required. HASMCs were cultured in complete medium at 37 °C in a humidified atmosphere containing 5% CO_2_. Cells in the logarithmic growth phase were used for all experiments. To evaluate the effect of CSE on HASMCs proliferation, cells were treated with different concentrations of CSE (0%, 0.2%, 0.4%, 0.6%, and 0.8%), and cell viability was determined using the CCK-8 assay.

Briefly, cells were washed with PBS, digested with trypsin for 1–3 minutes, and resuspended to obtain a single-cell suspension. Ten microliters of the suspension were used for counting, and the remaining cells were centrifuged at 1000 rpm for 5 minutes and resuspended in culture medium. Cells were seeded into 24-well plates at a density of 15,000 cells/well, with three replicates per group, and incubated overnight at 37 °C, 5% CO_2_. The next day, CSE solutions of the indicated concentrations were added (500 μL per well), and cells were cultured for 24 hours. After treatment, 50 μL of CCK-8 reagent was added to each well, and cells were incubated for 1.5 hours at 37 °C, 5% CO_2_. Subsequently, 100 μL of each reaction mixture was transferred into a 96-well plate (in triplicate), mixed thoroughly, and the optical density (OD) was measured at 450 nm and 620 nm using a microplate reader. Data were recorded for further analysis.

### 2.3. RNA extraction, library construction, and sequencing

Total RNA was extracted from～1x10^5^ cells using total RNA extraction Kit (DP431, TIANGEN, China), resulting 1.6–1.8 μg total RNA for each sample. The quality and quantity of the purified RNA were determined by measuring the absorbance at 260nm/280nm (A260/A280) using Ultrafine spectrophotometer (N50 touch, IMPLEN, Germany). The integrity of RNA was further verified by 1.0% agarose gel electrophoresis.

Oxford Nanopore Technologies (ONT) assays were performed by Wuhan Ruixing Biotechnology Co., Ltd (http://www.rxbio.cc). ONT library preparation steps with specific reagent volumes (9 μL RNA, 1 μL VNP primer, etc.) and barcoding kit (SQK-NBD114.96). Specifically, 500 ng total RNA was transferred to 0.2 ml PCR tubes and adjusted to a final volume of 9 μl with nuclease free water. Reactions were prepared (9 μl total RNA, 1 μl 10 μM VNP primer, 1 μl 10 mM dNTPs) and incubated for 5 min at 65°C, then snap cooled on a pre-chilled freezer block. Strand-switching buffer (4 μl 5x RT buffer, 1 μl RNaseOUT, 1 μl nuclease-free water, and 2 μl 10 μM strand-switching primer) was then added to the snap-cooled, annealed mRNA, and incubated at 42°C for 2 min. One μl of Maxima H Minus Reverse Transcriptase was added, and reactions were incubated at 42°C for 90 min, 85°C for 5 min, then held at 4°C. DNA library were set up for each cDNA (2 μl PR1 Primer, 2 μl PR2 Primer, 20 μl first-strand cDNA, 25 μl LongAmp Taq 2x master mix, 1 μl nuclease free water), and cycled for [3 min at 94°C] ×1 cycle, [15 s at 94°C, 15 s at 50°C, 2 min at 65°C] ×12 cycles, [10 min at 65°C] ×1 cycle, then held at 4°C. cDNA were added barcodes using the Native Barcode (NB01–96)(SQK-NBD114.96) following manufacturer’s guidelines.

Each barcoded DNA was purified using 1 × Ampure XP Beads, eluted in 20 μl of nuclease free water and quantified using Qubit. A total of 300 fmol of the adapter-ligated library was loaded onto a PromethION flow cell (vR10.4.4) and sequenced on a PromethION 2 Solo platform (Oxford Nanopore Technologies, Oxford, UK).

### 2.4. Long‑read sequencing data quality control, alignment, correction and Identification of transcripts

The raw Nanopore sequencing data in POD5 format were base-called into FASTQ files using Dorado (v0.8.2). Pychopper (v2.7.9; github.com/nanoporetech/pychopper) was then employed to trim reads and identify full-length transcripts. Quality filtering was performed with Chopper (v0.7.0; https://github.com/wdecoster/chopper) to retain only reads with a quality score ≥10 and length ≥50 bp for subsequent analysis.

Filtered reads were aligned to the Ensembl reference genome (GRCh38) using Minimap2 (v2.27-r1193) [23] in splice-aware mode with parameters ‘-ax splice -uf -k14’. We used the ﬂair [24] pipeline to identify the full-length of isoforms and ﬁltered them using the following criteria: (1) transcripts whose 5′ end was located within 100 bp from the TSS annotated by refTSS [25] and/or TSSclassiﬁer (“relaxed” or “strict”), which are based on the FANTOM CAGE (Cap Analysis of Gene Expression) peak [26], were extracted, (2) transcripts that have at least three full-length supporting reads (80% coverage and spanning 25 bp of the ﬁrst and last exons) in total with the “--stringent“option. Then we used SQANTI3 [27] with default parameters to filter out transcripts that are considered artifacts and to classify structural catogories.

### 2.5. Conversion of abundance estimates to transcripts per million (TPM)

To evaluate the accuracy of abundance estimation with transcript and gene resolution, we used the quasi-mapping mode of Salmon(v1.7.0) [28] to estimate the TPM for each transcript and gene in each of the sample data.

### 2.6. Differential gene and transcript expression analysis

We used DESeq2 (version 1.30.1) [29] to analyze the differences in transcripts and genes between the two groups. A statistical cutoff of Fold change > 2 and FDR < 0.05 was used to find differentially expressed genes (DEGs) and differential transcript expression (DETs).

### 2.7. Pathway enrichment analysis

Gene Ontology (GO) terms and KEGG pathways were identified using KOBAS(veision 2.0) [30]. Hypergeometric test and Benjamini-Hochberg FDR controlling procedure were used to define the enrichment of each term.

### 2.8. Differential alternative splicing analysis

SUPPA2(version 2.3) [31] was used for calling AS differences. For each identified AS event, the splicing level was defined by Percentage of Spliced-In (PSI) while the mean difference of splicing levels between two groups was measured by ΔPSI (dPSI) and by the P-value from independent T-tests against PSIs in the two groups. We screened out the difference AS with dPSI ≥ 0.05 and P value < 0.05 for analysis.

### 2.9. Analysis of alternative polyadenylation and the change in 3’UTR length

TAPAS [32] was used to identify analyze polyadenylation sites (PASs), alternative polyadenylation sites (APAs) and differential APA sites (Fold change ≥ 2; P value ≤ 0.05). We calculated the 3’UTR length value based on the genomic position difference between the last nucleotides of the longer and shorter 3’ UTR. We compared each pair of APAs in a given gene. When the logFC was negative, it indicates a 3’UTR shortening, and a positive logFC indicates a 3’UTR. lengthening. Fold change ≥ 2 was used for identification of confident change in 3’UTR length.

### 2.10. Transcription factor co-expression and target network analysis

To investigate potential regulatory relationships between transcription factors (TFs) and differentially expressed genes (DEGs), co-expression analysis was performed. Pearson correlation coefficients were calculated between the expression levels of differentially expressed TFs and all DEGs. TF-DEG pairs with an absolute correlation coefficient > 0.9 and a P value ≤ 0.01 were considered significantly co-expressed.

To further identify potential direct regulatory interactions, the target genes of the TFs of interest were retrieved from three publicly available databases: ENCODE (https://www.encodeproject.org/), TfbsDB (http://tfbsdb.systemsbiology.net/download), and TRRUST (https://www.grnpedia.org/trrust/). Genes annotated as targets in at least one of these databases were considered as known direct targets. Functional enrichment analysis of target genes was performed using Gene Ontology (GO) terms to identify overrepresented biological processes.

### 2.11. Statistical analysis

All plots were done in R (v 4.2.3) as implemented in RStudio in addition to pattern diagrams and stacked bar chart. Data are presented as means ± standard error of the mean (SEM). Statistical difference between two groups was calculated by Student’s t-test.

## 3. Result

### 3.1. A Comprehensive Full-Length Transcriptome Landscape of Cigarette Smoke-Exposed HASMCs

**Fig 1:**
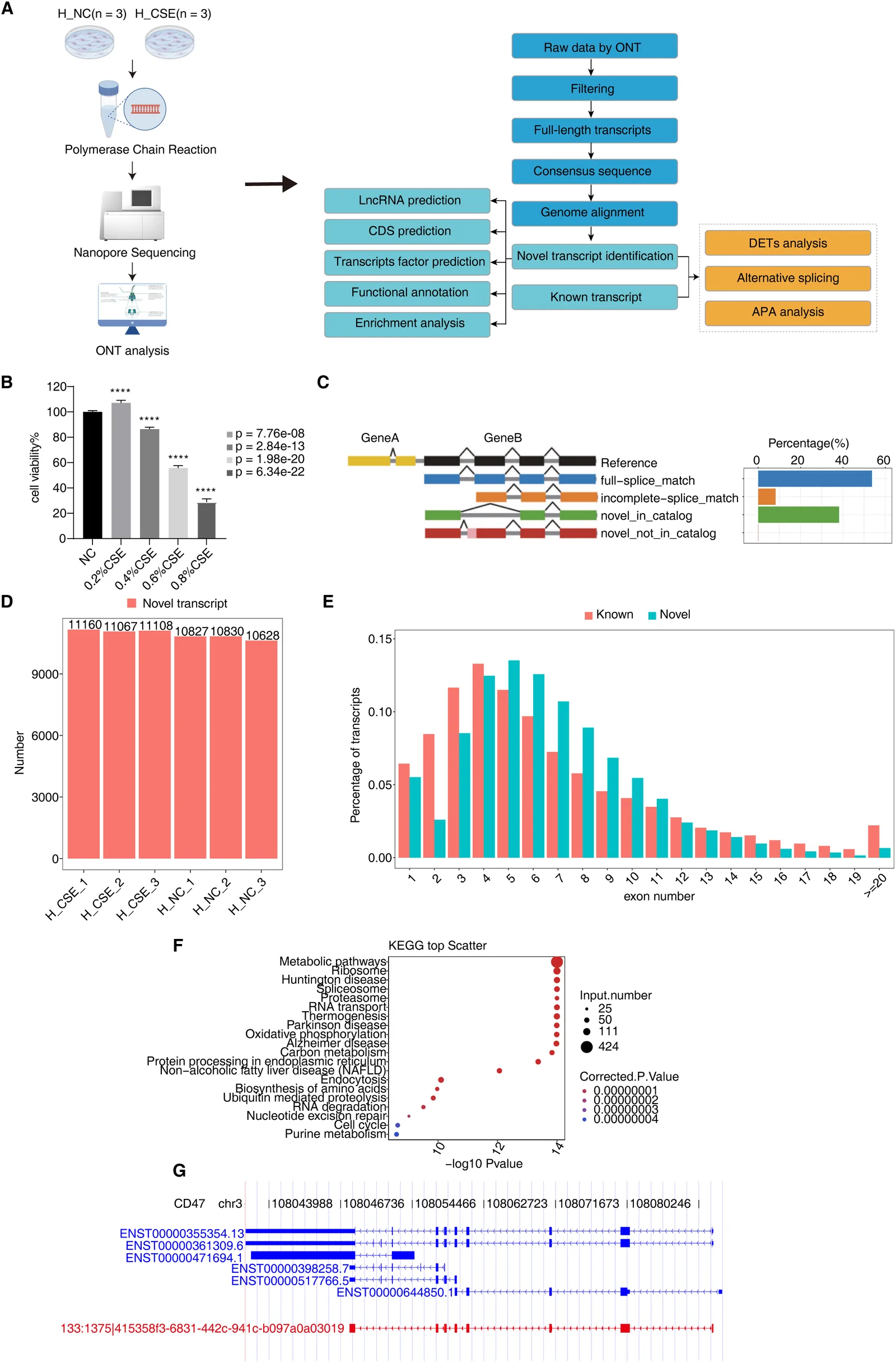
Full-length transcript identification and characterization in cigarette smoke-exposed and control HASMCs. A. Illustration of the the experimental strategy of this study B. Bar plot of the percentage of the cell viability of CSE-treated HASMCs when compared with that of the control HASMCs. Two-tailed Student’s t-test was performed, and **** indicates P value ≤ 0.0001. The the exact p-values for each comparison was listed at the upper-right corner. C. The proportion of structural categories of isoforms in CSE-treated and control HASMCs. FSM (full splice match), indicating the reference and query isoform have the same number of exons, and each internal junction agrees; ISM (incomplete splice match), indicating the query isoform has fewer 5’ exons than the reference, but each internal junction agrees; NIC (novel in catalog), indicating the query isoform does not have an FSM or ISM match, but is using a combination of known donor/acceptor sites; NNC (novel not in catalog), indicating the query isoform does not have an FSM or ISMmatch, and has at least one donor or acceptor site that is not annotated. D. Bar plot of the number of the detected novel isoforms in each sample. E. Bar plot of the percentage of transcripts containing different number of exons. F. KEGG enrichment analysis of the novel transcripts. G. The representative of the identification of a novel transcript in CD47. The structure of known transcripts are depicted in blue, and the novel transcript in red.

The experimental strategy of this study is outlined in Fig 1A. While some early studies reported that cigarette smoke extract (CSE) could enhance the proliferation and viability of human aortic smooth muscle cells (HASMCs), they also documented an inverse correlation at higher CSE concentrations [21,22]. This inhibitory effect at higher doses was later confirmed in a subsequent study [20]. Consistent with the latter, our experiments using 0.2%−0.8% CSE revealed only a marginal increase in cell viability at 0.2% CSE, followed by a clear, dose-dependent decrease at concentrations ranging from 0.4% to 0.8% (Fig 1B).

To elucidate the molecular mechanisms underlying the CSE-induced decline in HASMC viability, we performed full-length transcriptome sequencing on the Oxford Nanopore Technologies (ONT) platform. Based on the dose-response curve of the CSE cytotoxicity (Fig 1B), the midpoint concentration (0.6%) was selected for subsequent transcriptomic analysis. We analyzed polyadenylated mRNA and lncRNA from control and 0.6% CSE-treated HASMCs, with three biological replicates per group. After filtering, we obtained 4.8–5.8 million reads per sample, of which 4.2–5.0 million were full-length (**S1**A **Fig in S1 File**). Characterization of all detected full-length transcripts identified 16,422 full-splice matches (FSM), 2,506 incomplete-splice matches (ISM), 11,203 novel in catalog (NIC) isoforms, and 19 novel not in catalog (NNC) isoforms (Fig 1C) (S1 Table). CSE treatment led to slightly more novel isoforms (Fig 1D).

Analysis of exon numbers revealed that novel transcripts predominantly contained 4–7 exons per gene, whereas known transcripts typically possessed 3–6 exons (Fig 1E). Functional annotation of the 11,222 novel full-length transcripts indicated that 9,803 and 9,527 had matches in the NR and UniProt databases, respectively, suggesting that at least 87.66% originated from protein-coding genes (S1B Fig in S1 File). KEGG pathway analysis showed that these novel transcripts were enriched in metabolic pathways (424 transcripts), as well as in ribosomal (111) and spliceosomal (63) components. A substantial fraction was also associated with neurodegenerative diseases (Fig 1F). Consistent with this, KOG annotation identified 143 and 253 novel isoforms involved in RNA processing/modification and translation, respectively (S1C Fig in S1 File). Furthermore, Pfam domain analysis indicated that a large proportion of novel isoforms contained zinc finger or RNA recognition motif (RRM) domains, implicating potential RNA-binding activity (S1D Fig in S1 File). Representative isoform structures for two genes, CD47 and ACTRA1, are displayed in Fig 1G and S1E in S1 File, respectively. CD47 expression change has been implicated in smoking response [33,34] Collectively, these findings highlight the prevalence and potential functional significance of the novel transcripts identified in our study.

### 3.2. Functional Annotation of Differentially Expressed Transcripts and Genes in Cigarette Smoke-Exposed HASMCs

**Fig 2:**
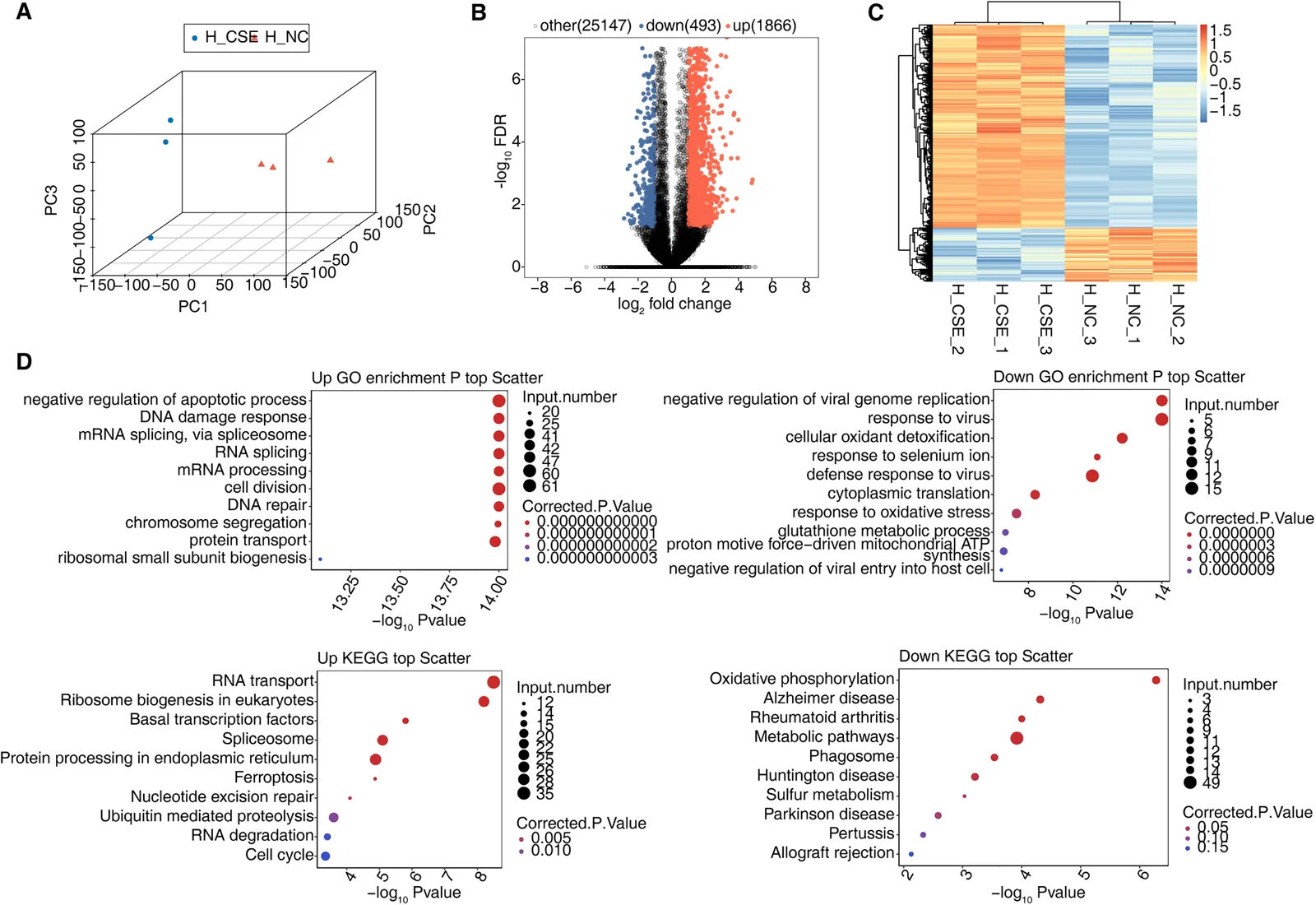
Functional annotation of differentially expressed transcripts (DET) in cigarette smoke-exposed and control HASMCs. A. Principal component analysis (PCA) based on TPM value of all detected transcripts. The ellipse and triangle for each group is the confidence ellipse and triangle. B. Volcano plot presenting all DETs between CSE-treated and Control (NC) samples with DESeq2. (FC > 2 or < 0.5, FDR < 0.05) C. Heatmap plot of the expression of DETs across all samples. D. The top 10 most enriched GO and KEGG terms (biological process) were illustrated for up and down-regulated DETs in the CSE vs NC groups.

Principal component analysis (PCA) revealed a clear separation between CSE-treated and control HASMCs in both transcript and gene expression profiles, indicating systematic alterations in transcriptional composition upon CSE exposure (Fig 2A and S2A in S1 File).

To systematically characterize the dynamic changes in gene and transcript expression induced by CSE, we performed differential expression analysis using DESeq2 on the ONT full-length transcriptome data. We identified 1,866 upregulated and 493 downregulated transcripts (Fig 2B, S2 Table), along with 1,214 upregulated and 318 downregulated genes (S2B Fig in S1 File). The CSE-induced expression changes were highly consistent across experimental replicates (Fig 2B and S3B in S1 File).

Gene Ontology biological process (GO-BP) analysis indicated that upregulated transcripts were significantly enriched in processes such as negative regulation of apoptosis, cell division, and chromosome segregation (Fig 2D). In contrast, downregulated transcripts were strongly associated with antiviral and innate immune responses, cellular oxidant detoxification, response to selenium, oxidative stress response, glutathione metabolism, and proton motive force-driven mitochondrial ATP synthesis (Fig 2E). KEGG pathway analysis further highlighted upregulation of genes involved in ferroptosis and cell cycle, alongside downregulation of oxidative phosphorylation-related genes (Fig 2F–G). Functional enrichment based on differentially expressed genes yielded similar results to those from transcript-level analysis (S2D–G Fig in S1 File).

In summary, our transcriptomic analysis reveals that CSE exposure induces widespread and systematic changes in the HASMC transcriptome. The functional enrichment of these differentially expressed transcripts and genes strongly correlates with the observed suppression of cell viability. These findings collectively underscore that CSE-induced HASMC damage is accompanied by the coordinated involvement of activated processes like apoptosis, ferroptosis and cell cycle dysregulation, alongside the critical suppression of protective mechanisms, including innate immune response, oxidative phosphorylation and glutathione-mediated antioxidant defense.

### 3.3. Identification and Functional Analysis of Key Genes and Transcripts in Cigarette Smoke-Exposed HASMCs

**Fig 3:**
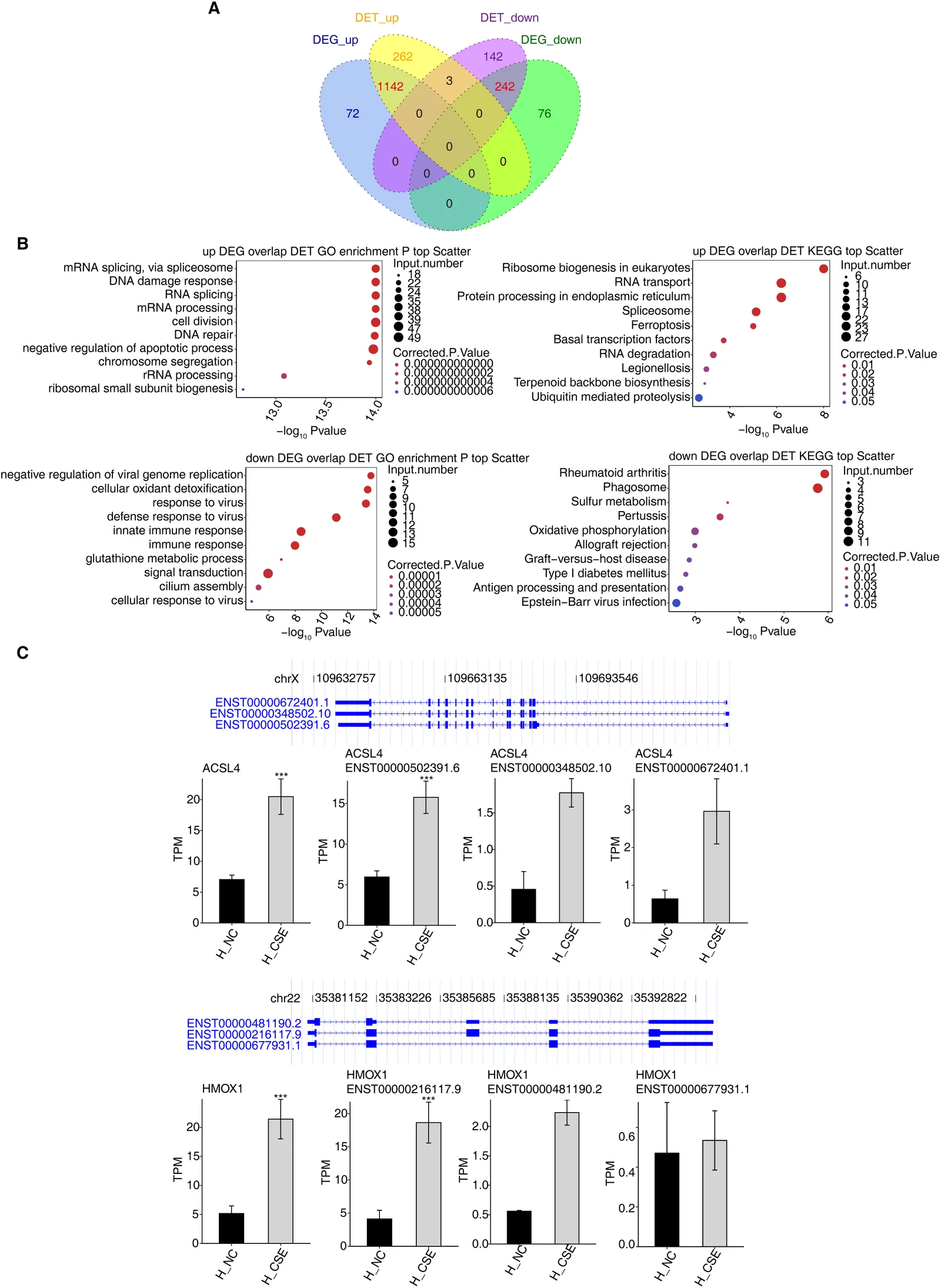
Integrated functional analysis of overlapping genes among the up- and down-regulated DET-containing genes and DEGs. A. Venn diagram showing the overlap among up- and downregulated genes containing DETs, DEGs. B. Scatter plots of the the top 10 most enriched GO biological process and KEGG pathways for the overlapped DET and DEG. C. The structure of the detected transcripts and statistical difference of the expression of ACSL4 and HMOX1 at the gene and transcript levels. Error bars represent mean ± SEM. ***: P value ≤ 0.001.

To identify core regulatory genes and transcripts, we integrated the results from differentially expressed genes (DEGs) and differentially expressed transcripts (DETs), identifying a total of 1384 overlapping genes (Fig 3A). Among these, the majority of upregulated DET-containing genes (81.3%) were also upregulated DEGs, while 63.0% of downregulated DET-containing genes corresponded to downregulated DEGs (Fig 3A), indicating that CSE-induced alterations in the transcriptomic landscape predominantly occur at the transcriptional level.

Gene Ontology (GO) analysis revealed that the top 10 enriched biological processes among the overlapping genes closely mirrored those associated with DETs. These included upregulated processes such as ferroptosis, cell division, and negative regulation of apoptosis, as well as downregulated processes including innate immune response, cellular oxidant detoxification, glutathione metabolism, and oxidative phosphorylation (Fig 3B–C).

Within the ferroptosis-related genes and transcripts upregulated in HASMCs following cigarette smoke exposure, most were identified as ferroptosis drivers, including ACSL3, ACSL4, ATG5, HMOX1, NCOA4, SAT1, and TFRC. Notably, for certain DEGs such as ACSL4, ATG5, and TFRC, all detected transcripts were upregulated. In contrast, for other genes, only specific transcripts showed increased expression. This observation underscores the significant role of post-transcriptional regulation in modulating the expression levels of individual isoforms.

### 3.4. Differential Alternative Polyadenylation Landscape in Cigarette Smoke-Exposed HASMCs

**Fig 4:**
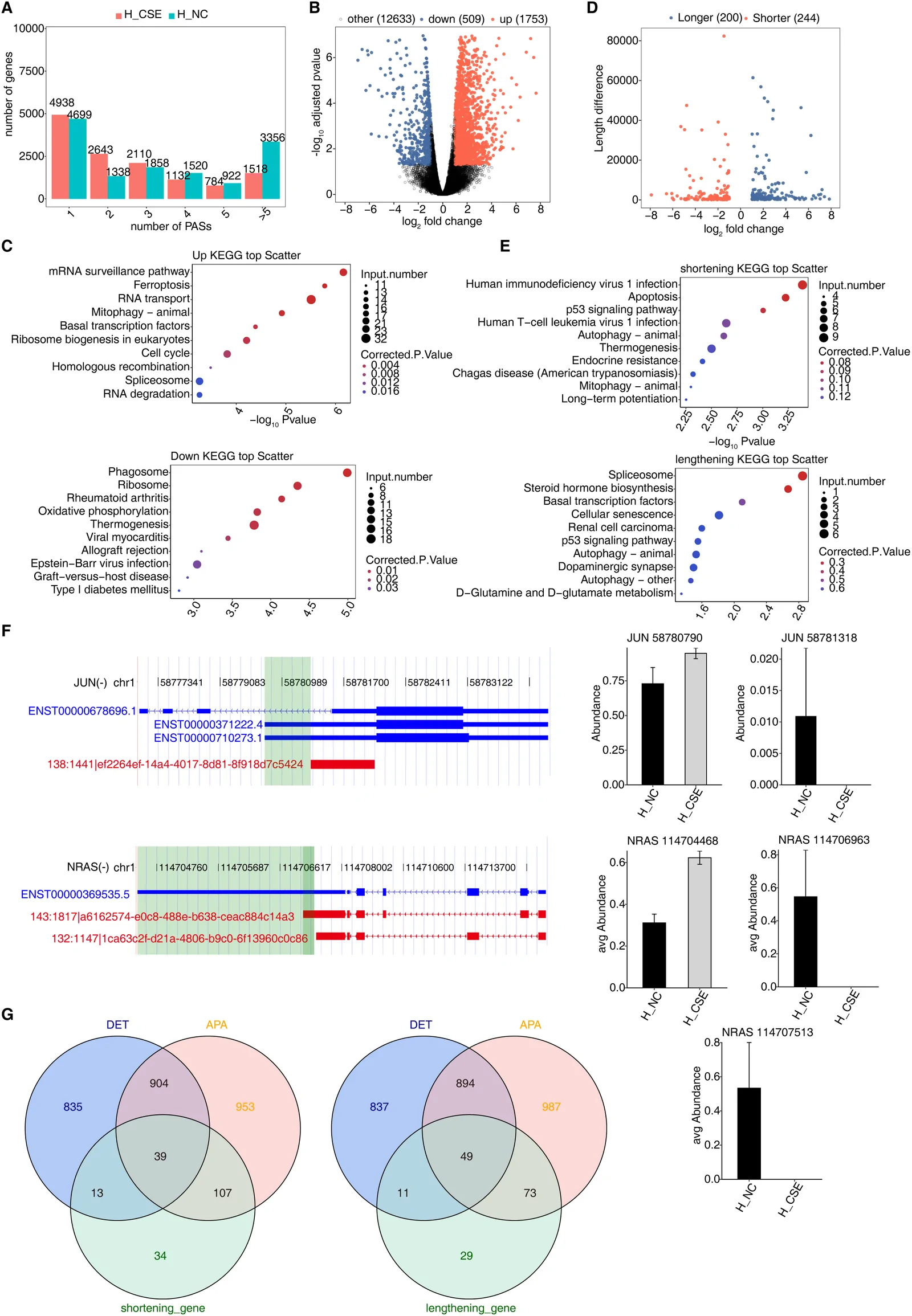
Analysis of the differential polyadenylation and the change in 3’UTR length in the cigarette smoke-exposed and control HASMCs. A. Bar plots of genes containing different number of PASs B. Volcano plot presenting all DEAPAs between CSE-treated and Control (NC) samples with DESeq2. (FC > 2 or < 0.5, FDR < 0.05) C. Scatter plot of the top 10 most enriched KEGG pathways were illustrated for up and down-regulated APA genes. D. Volcano plot presenting the 3’UTR length change between CSE-treated and Control (NC) samples with DESeq2. (FC > 2 or < 0.5, FDR < 0.05). Y-axis indicates the length difference. E. Scatter plot of the top 10 most enriched KEGG pathways were illustrated for genes containing the lengthing and shortening 3’UTRs. F. The structures and expression changes of two apoptosis genes JUN and NRAS containing longer 3’UTR in the CSE-treated group. Known transcripts are depicted blue, and the novel transcripts in red. G. Venn plot of the overlap among DET, APA and 3’-UTR shortening and lengthening genes.

We systematically analyzed alternative polyadenylation (APA) changes in long-read transcripts from cigarette smoke-exposed human aortic smooth muscle cells (HASMCs). Statistical analysis of polyadenylation sites (PASs) per gene revealed that control transcriptomes expressed more genes with 1–3 PASs, whereas CSE-treated transcriptomes exhibited a higher proportion of genes with ≥4 PASs (Fig 4A), indicating that CSE promotes the usage of alternative polyadenylation sites.

Differential APA analysis identified 1753 significantly increased and 509 decreased APA events, reflecting a pronounced shift toward increased APA usage (Fig 4B). Genes harboring differential APAs were significantly enriched in GO biological processes such as ferroptosis, mitophagy, and cell cycle, as well as the KEGG pathway “oxidative phosphorylation” (Fig 4C)—consistent with the functional profiles observed for differentially expressed transcripts (Fig 2). This similarity was further supported by the finding that approximately half of all differential APA events overlapped with differentially expressed transcripts (Fig 4G).

Changes in APA resulted in 247 genes with shorter 3′UTRs and 200 genes with longer 3′UTRs (Fig 4D). Genes with 3′UTR lengthening were enriched in apoptosis, the p53 signaling pathway, autophagy, and mitophagy, while those with 3′UTR shortening were associated with cellular senescence, the p53 signaling pathway, and autophagy (Fig 4E). Illustrating these trends, several apoptotic genes—including CTSC, FAS, ATM, NRAS, JUN, AKT3, and MAPK8—displayed 3′UTR lengthening in CSE-exposed HASMCs (Fig 4F and S4 in S1 File).

### 3.5. Differential Alternative Splicing Landscape in Cigarette Smoke-Exposed HASMCs

**Fig 5:**
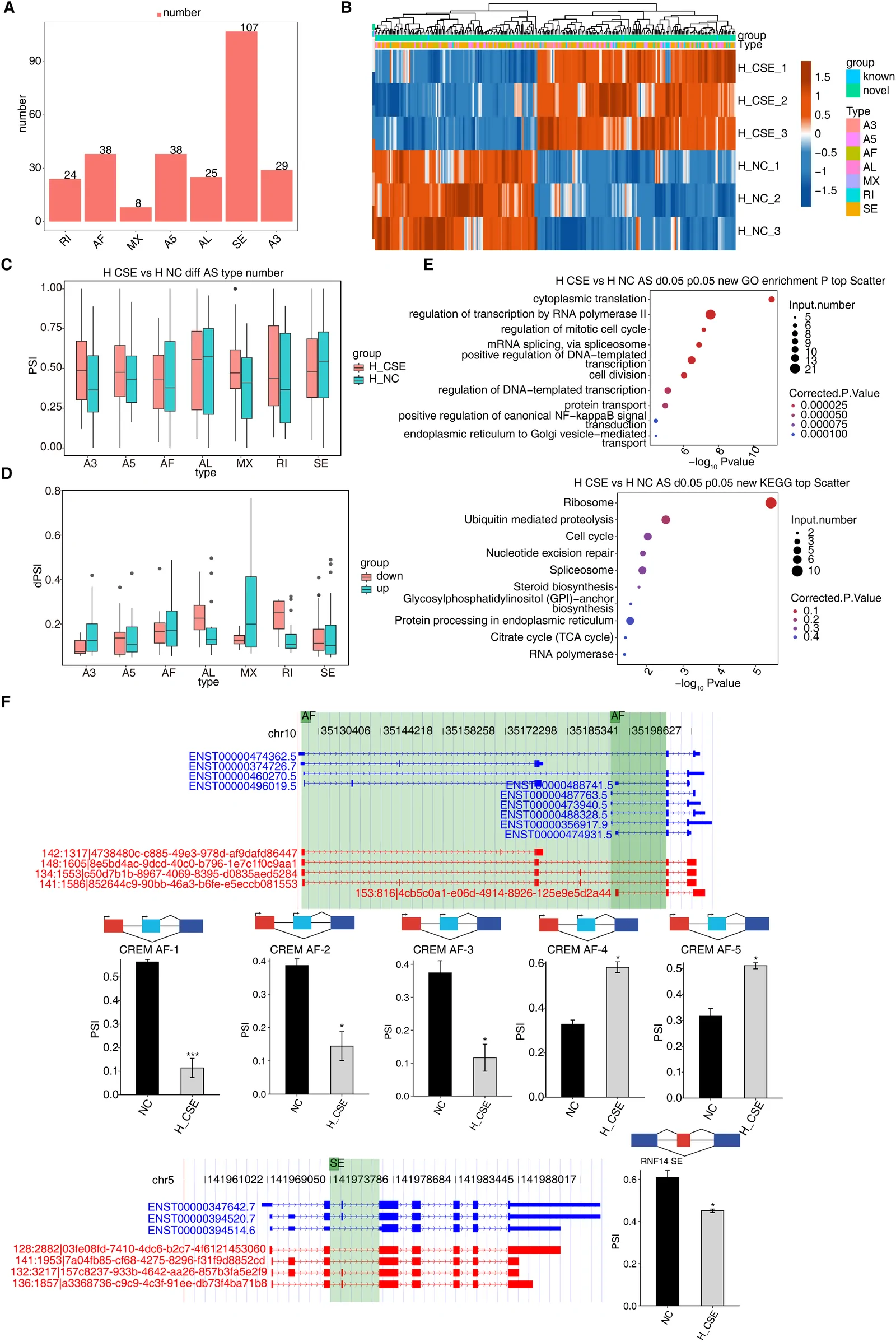
The dysregulation of alternative splicing (AS) events in cigarette smoke-exposed HASMCs. A. The bar plot showing the number of differentially spliced alternative splicing events. B. Heatmap plot of the PSI calue of all differentially spliced alternative splicing events C. The box plot showing the PSI value of differentially spliced alternative splicing events. The black dot indicates individual outlier event. No statistical difference was evident between CSE-exposed and control samples. D. The box plot showing the dPSI value of differentially spliced alternative splicing events. Black dots indicate individual outlier events. No statistical difference was evident between CSE-exposed and control samples. E. The top 10 most enriched GO biological process and KEGG pathways were illustrated for genes of DASG. F. Representative illustration of the alternative splicing events, CREM with 5 alternative first exon events (AF), and RNF14 with one exon skipping event (SE). The structure of related transcript isoforms identifying these alternative splicing events were shown, Known transcripts are depicted blue, and the novel transcripts in red. The bar plot showing the PSI of each alternative splicing event (ASE) and the illustration on the top of the bar showing the corresponding alternative splicing type. We compute significance by using the empirical method of Suppa2. *: P value < 0.05.

A systematic analysis of alternative splicing in CSE-treated and control HASMCs identified 269 differential alternative splicing events. Exon skipping (SE) was the predominant type of alternative splicing event (Fig 5A). Using SUPPA2, we calculated the percent spliced-in (PSI) values for each event, which revealed highly consistent splicing ratio changes between groups (Fig 5B). Comparison of PSI values across splicing types showed that alternative 3′ splice site (A3) events exhibited a relatively larger median PSI difference between conditions compared to other splicing types (Fig 5C). Furthermore, evaluation of splicing change magnitude using dPSI values indicated that alternative last exon (AL) events displayed the greatest variation (Fig 5D).

Functional enrichment analysis showed that differentially alternatively spliced genes were significantly associated with GO biological processes such as regulation of the mitotic cell cycle, cell division, and positive regulation of canonical NF-kappaB signal transduction, as well as the KEGG pathway “cell cycle” (Fig 5E). These results underscore the role of alternative splicing in mediating cigarette smoke-induced dysregulation of cell proliferation.

We further visualized CSE-disrupted splicing events in genes involved in mitotic cell cycle regulation, including CDC23, ANAPC10, FBXO5, DLGAP5, GMNN, and CDK5RAP3 (S5B Fig in S1 File). Notably, splicing of transcription factors was particularly susceptible to CSE-induced alterations, suggesting that splicing dysregulation may propagate transcriptomic changes by shifting the isoform balance of key regulators (Fig 5E). Differential splicing events in transcription factors such as RNF14, PCGF5, CDK1, CREM, and FOSL1 are shown in Fig 5F and S5B in S1 File, along with the corresponding transcript isoform structures supporting these events, further validating the reliability of our findings.

### 3.6. Cross-regulatory network of DET, AS, and DEG in Cigarette Smoke-Exposed HASMCs

**Fig 6:**
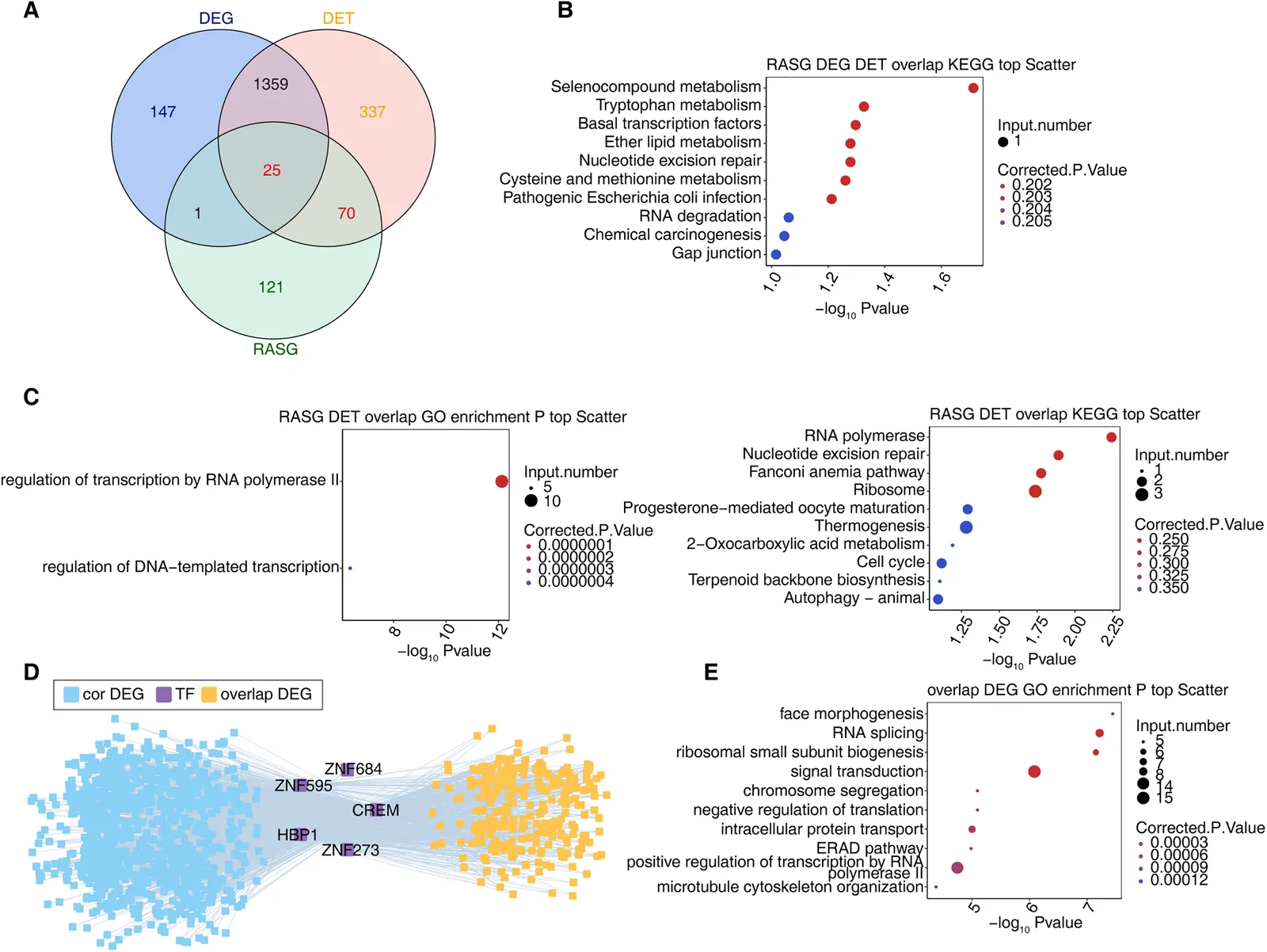
Cross-regulatory network of DET, AS, and DEG in cigarette smoke-exposed HASMCs. A. Venn diagram showing the overlap among genes containing DETs, ASEs and DEGs. B. Scatter plot of the KEGG enrichment of the 25 genes shared by DET, DEG and RASG. C. Scatter plot of the GO and KEGG enrichment of the 70 overlapped genes only shared by DET and RASG. D. The co-expression network constructed by Cytoscape shows the Pearson correlation between key transcription factors (TFs) and differentially expressed genes (DEGs). Those also harboring the binding sites of CREM and/or HBP1 at their promoters were depicted in orange, while those lacking the binding sites were depicted in blue. E. Scatter plot of the GO enrichment of the CREM and HBP1-targeted DEGs.

We then integrated analyses of differentially expressed genes (DEGs), genes containing differentially expressed transcripts (DETs), and genes harboring differential alternative splicing events in CSE-exposed HASMCs. This multi-level approach identified 25 genes that were concurrently altered at the gene expression, transcript expression, and alternative splicing levels (Fig 6A). Functional enrichment analysis revealed that these genes were most significantly associated with selenocompound metabolism (Fig 6B).

Notably, genes with differential alternative splicing (RASGs) exhibited greater overlap with DET genes than with DEGs. In particular, DET genes that did not overlap with DEGs showed a significantly stronger tendency to co-occur with RASGs (70; Fig 6A), suggesting that alternative splicing serves as an important mechanism underlying transcript-level expression changes. Furthermore, the subset of DET genes that also belonged to RASGs was highly enriched in transcription-related regulatory functions (Fig 6C), reinforcing the role of alternative splicing in reshaping the transcriptional landscape in response to cigarette smoke exposure.

### 3.7. Core Regulatory Network of Transcription Factors Associated with Differential Expression and Alternative Splicing

To investigate whether post-transcriptional dysregulation may propagate through regulatory networks, we focused on transcription factors that were both differentially expressed at the transcript level and harbored alternative splicing events (overlap between DET and RASG). Among the 10 genes enriched in RNA polymerase II transcription regulation (Fig 6C), five transcription factors were identified: CREM, HBP1, ZNF273, ZNF595, and ZNF684.

We constructed co-expression networks by calculating Pearson correlation coefficients between the expression levels of these five transcription factors and all differentially expressed genes (DEGs). Using stringent thresholds (|correlation| > 0.9, adjusted P ≤ 0.01), we identified 1,281 out of 1,532 DEGs that were significantly correlated with at least one of the five transcription factors (S3 Table). Notably, CREM exhibited co-expression with 1,150 DEGs, while HBP1 co-expressed with 902 DEGs. In contrast, ZNF595, ZNF273, and ZNF684 showed co-expression with 181, 1, and 3 DEGs, respectively (Fig 6D; S3 Table).

To further elucidate potential direct regulatory relationships, we queried known transcription factor target databases (e.g., ENCODE, TfbsDB, TRRUST). Among the 1,150 CREM-co-expressed genes, 54 were annotated as direct targets of CREM; similarly, among the 902 HBP1-co-expressed genes, 160 were annotated as direct targets of HBP1 (S4 Table). No direct target annotations were available for ZNF273, ZNF595, or ZNF684. Gene Ontology (GO) analysis of these direct target genes revealed significant enrichment in biological processes such as “chromosome segregation”, “microtubule cytoskeleton organization”, “cell division”, “defense response to virus”, which may explain the CSE injury (Fig 6E) (S5 Table).

These findings suggest that CSE-induced alternative splicing of CREM and HBP1—both of which exhibited splicing changes in our analysis (Fig 5F)—may contribute to downstream transcriptional reprogramming by directly regulating genes involved in cell cycle progression. This provides initial evidence supporting a feed-forward cascade model in which post-transcriptional alterations in key transcription factors propagate through their target gene networks to influence broader cellular phenotypes.

It should be noted that these findings represent correlative associations; causal relationships require functional validation.

## 4. Discussions

Our study addresses a long-standing paradox in the literature regarding the impact of cigarette smoke on VSMC biology. While some reports indicate a pro-proliferative effect, others, including our own data, clearly demonstrate a potent cytotoxic response at higher concentrations. We first systematically confirmed this dual effect, showing a marginal increase in viability at a very low CSE concentration (0.2%) followed by a robust, dose-dependent inhibition at higher doses (0.4%−0.8%). This critical observation allowed us to focus on elucidating the molecular mechanisms of the cytotoxic phase, for which we provided a comprehensive, isoform-resolved map using ONT long-read sequencing.

The full-length transcriptome analysis revealed an unexpected layer of complexity in the HASMC response to CSE: we identified 11,222 novel transcripts that substantially expand the known transcriptome. Functional annotation suggests their involvement in diverse cellular processes: enrichment in metabolic pathways implicates them in CSE-induced metabolic reprogramming, while their presence among ribosomal and spliceosomal components points to roles in protein synthesis and RNA processing. Notably, these novel transcripts were enriched in pathways associated with Huntington’s, Parkinson’s, and Alzheimer’s diseases, suggesting shared molecular mechanisms—such as mitochondrial dysfunction, oxidative stress, and impaired autophagy—between smoking-induced vascular pathology and neurodegeneration. Furthermore, Pfam domain analysis revealed that many novel isoforms contain zinc finger or RRM domains, indicating potential RNA-binding activity and possible involvement in post-transcriptional regulatory networks.

Beyond the identification of thousands of novel transcripts, our functional data paint a coherent picture of a cell under severe metabolic and genotoxic stress. The upregulation of ferroptosis and cell division genes, coupled with the simultaneous suppression of oxidative phosphorylation, glutathione metabolism, and antiviral defense pathways, reveals a complex transcriptional response to CSE exposure (Fig 2). The increased expression of cell-cycle genes, however, does not reflect active proliferation given the observed reduction in cell viability. Rather, it likely indicates dysregulated cell-cycle checkpoints and mitotic stress—a state in which cell-cycle progression is aberrantly initiated but cannot be successfully completed. This interpretation is consistent with the enrichment of genes involved in chromosome segregation and mitotic regulation among the differentially expressed transcripts. Concurrently, the suppression of oxidative phosphorylation and glutathione metabolism points to a fundamental collapse in energy production and antioxidant defense. Together, these perturbations converge on the activation of specific cell death pathways, including ferroptosis.

Consistent with our findings, ferroptosis has been increasingly implicated in the pathogenesis of smoke-related diseases [35–37]. For instance, cigarette smoke can trigger ferroptosis in human bronchial epithelial cells through ROS accumulation and iNOS signaling, while MFG-E8 has been identified as a key suppressor of this process in the same cell type [37,38]. Furthermore, the work by Sampilvanjil et al. directly demonstrated CSE-induced ferroptosis in VSMC [39]. Moving beyond these prior observations, our study provides a critical advancement by delineating the alterations in ferroptosis-related genes at the full-length transcript level. This high-resolution view is essential for future efforts to manipulate the ferroptotic pathway with greater precision, as it reveals the specific transcript isoforms that are dysregulated, thereby offering more targeted therapeutic opportunities.

The most significant advances from our work lie in the detailed dissection of the post-transcriptional regulatory layer. Consistent with the previous finding of the widespread 3′ UTR lengthening in blood transcriptome associated with smoking [15], we for the first time demonstrated the similar result from the CSE-treated HASMCs, indicating that smoking is a powerful regulator of 3’ UTR length. We showed that the 3’UTR lengthening/shortening is occurred particularly in genes controlling apoptosis and p53 signaling, autophagy and cellular senescence, which are important mechanisms in mediating smoking toxicity [40–45]. The 3’UTR length- regulated genes include NRAS, JUN, CTSC, AKT3, MAPK8, ATM and FAS, several of which have been implicated in smoke-induced injury [46–48]. Furthermore, the discovery of 269 differentially spliced genes, heavily enriched for cell cycle regulators and, crucially, transcription factors, suggests a powerful feed-forward mechanism. We hypothesize that CSE-induced alternative splicing of master transcription factors (e.g., CREM, HBP1, FOSL1, CDK1, RNF14) may act as an amplification cascade, whereby key regulators could influence hundreds of downstream targets, globally reprogramming the cellular transcriptome and reinforcing the pathological phenotype. While this hypothesis requires experimental validation, it offers a compelling framework for understanding how initial post-transcriptional events might propagate through the regulatory network.

Several limitations of this study should be acknowledged. First, sequencing was performed at a single CSE concentration (0.6%). While this concentration represents the midpoint of the cytotoxic phase based on our dose-response data, future studies examining concentration-dependent isoform changes across the full range of CSE concentrations will be valuable to fully elucidate the molecular transition from proliferation promotion to cytotoxicity. Second, our study used a single immortalized cell line without validation in primary cells, animal models, or clinical samples. While this controlled system enabled comprehensive transcriptomic profiling, the translational relevance of our findings—particularly the novel isoforms and splicing events—requires confirmation in more complex systems such as smoke-exposed mouse aortas or vascular tissues from human smokers. Third, as a discovery-oriented study, our work does not include functional validation of specific isoforms or splicing events. However, the comprehensive catalog of isoform-level changes we provide offers a rich resource for future hypothesis-driven investigations. Targeted experiments such as isoform-specific overexpression, splice-switching antisense oligonucleotides, or 3’UTR reporter assays will be necessary to establish the functional consequences of the specific events identified here. Finally, while our bioinformatic characterization suggests that the majority of novel transcripts are protein-coding (87.66% with database matches), experimental validation—such as RT-PCR with Sanger sequencing or proteomic confirmation—will be essential to confirm their existence and coding potential. Similarly, the observed 3’UTR remodeling suggests—but does not prove—that miRNA or RBP binding may be altered; mechanistic studies (e.g., luciferase reporter assays, CLIP-seq) are needed to confirm functional consequences.

In conclusion, our research reconciles conflicting phenotypic observations by linking them to a unified transcriptomic narrative. We demonstrate that the cytotoxic effect of high-concentration CSE is not a simple outcome of gene dysregulation but is the result of a sophisticated and multi-layered post-transcriptional alterations, including the expression of thousands of novel transcripts, widespread APA-mediated 3’UTR remodeling, and alternative splicing of key regulatory factors. This work fundamentally shifts the understanding of smoking-induced vascular damage from a gene-centric view to an isoform-centric paradigm, revealing a previously underappreciated layer of regulatory mechanisms and potential therapeutic targets for combating smoking-related cardiovascular disease.

## Supporting information

S1 File Supplementary figures. (DOCX)

S1 Table The expression level of novel transcripts. (XLTX)

S2 Table Differentially expressed transcripts between CSE-treated and control HASMC cells. (XLTX)

S3 Table DEGs co-expressed with CREM, HBP1, ZNF595, ZNF273 and ZNF684. (XLSX)

S4 Table The overlap between the co-expressed DEGs (Table S3) and the annotated direct targets of CREM and HBP1. (XLSX)

S5 Table GO enrichment of the overlapped genes in Table S4. (XLTX)
