# Supplementary material for: A comprehensive full-length transcriptome landscape of cigarette smoke-exposed HASMCs reveals extensive remodeling of mRNA isoforms and regulatory networks
Source: PLoS One. 2026 Sep 21;21(9):e0354604. doi: 10.1371/journal.pone.0354604 (PMC13592704; doi:10.1371/journal.pone.0354604)
Supplement: Supplementary file 1 [file pone.0354604.s001.docx]

**Supplementary Figures**

**S1 Fig. Full-length transcript identification and characterization in cigarette smoke-exposed and control HASMCs (related to Fig 1).**

A.Bar plot of the statistics of the long-read sequencing results of this study, including clean sequences, full length sequences and uniquely mapped sequences.

B.Bar plot of the number and percentage of annotation of the novel transcripts.

C.KOG annotation of novel transcripts.

D.Pfam annotation of novel transcripts.

H.The representative of the identification of a novel transcript in ACTRA1. The structure of known transcripts are depicted in blue, and the novel transcripts in red.

**S2 Fig. Functional annotation of differentially expressed genes (DEG) in cigarette smoke-exposed and control HASMCs.**

A. Principal component analysis (PCA) based on TPM value of all detected genes. The ellipse and triangle for each group is the confidence ellipse and triangle.
B. Volcano plot presenting all DETs between CSE-treated and Control (NC) samples with DESeq2. (FC > 2 or < 0.5, FDR < 0.05)
C. Heatmap plot of the expression of DEGs across all samples.
D. The top 10 most enriched GO and KEGG terms (biological process) were illustrated for up and down-regulated DEGs in the CSE vs NC groups.

 **S3 Fig. The structure of the detected transcripts and statistical difference of the expression of NCOA4, SAT1, ACSL3 and ATG5 at the gene and transcript levels (related to Fig 3).** The expression of some transcripts of NCOA4 and SAT1 were not shown. Error bars represent mean ± SEM. ***: P value ≤ 0.001. The structure of known transcripts are depicted in blue, and the novel transcripts in red.

**S4 Fig. The structures and expression changes of three apoptosis genes FAS, MAPK8 and CYSC containing longer 3’UTR in the CSE-treated group**. Known transcripts are depicted blue, and the novel transcripts in red. (related to Fig 4)

**S5 Fig. The dysregulation of alternative splicing (AS) events in cigarette smoke-exposed HASMCs. (related to Fig 5)**

A. The bar plot showing the number of all spliced alternative splicing events.
B. Representative illustration of the alternative splicing events. The structure of related transcript isoforms identifying these alternative splicing events were shown, Known transcripts are depicted blue, and the novel transcripts in red. The bar plot showing the PSI of each ASE and the illustration on the top of the bar showing the corresponding alternative splicing type. We compute significance by using the empirical method of Suppa2. *: P value < 0.05.
