# Supplementary material for: A comprehensive full-length transcriptome landscape of cigarette smoke-exposed HASMCs reveals extensive remodeling of mRNA isoforms and regulatory networks
Source: PLoS One. 2026 Sep 21;21(9):e0354604. doi: 10.1371/journal.pone.0354604 (PMC13592704; doi:10.1371/journal.pone.0354604)
Supplement: Supplementary file 2 [file pone.0354604.s007.docx]

This study employs Oxford Nanopore long-read sequencing to systematically analyze the impact of cigarette smoke extract (CSE) on the full-length transcriptome of human aortic smooth muscle cells (HASMCs), revealing CSE-induced transcript isoform remodeling, alternative polyadenylation (APA), and alternative splicing (AS) events. The research design is sound, the data are comprehensive, and the work demonstrates a degree of novelty. However, several areas require refinement, including methodological descriptions, ethical statements, figure correspondence, and discussion depth.

1. Lines 91-92: Please cite the reference for the CSE preparation method.
2. Lines 92-95: Please provide the rationale for using the "Huanghelou" cigarette brand and discuss whether its specific composition is representative.
3. Lines 106-111: Please specify the source of the cell line and include an ethical statement.
4. Lines 124-146: Please provide additional methodological details regarding RNA extraction and ONT library preparation/sequencing.
5. In Figure 1B, it is recommended to annotate the specific p-values.
6. There is some inconsistency between the description in the main text and the data presented in Figure 1. Please verify and ensure consistency.
7. In the Discussion, please briefly address the potential functions of the newly identified transcripts in the cellular response to CSE.
8. Some statements in the text are overly definitive. A more nuanced phrasing is recommended.
9. Please verify all abbreviations used throughout the manuscript.
