# Supplementary material for: A comprehensive full-length transcriptome landscape of cigarette smoke-exposed HASMCs reveals extensive remodeling of mRNA isoforms and regulatory networks
Source: PLoS One. 2026 Sep 21;21(9):e0354604. doi: 10.1371/journal.pone.0354604 (PMC13592704; doi:10.1371/journal.pone.0354604)
Supplement: Supplementary file 3 [file pone.0354604.s008.docx]

****Manuscript ID:**** PONE-D-25-67222

****Title:**** A Definitive Full-Length Transcriptome Landscape of Cigarette Smoke-Exposed HASMCs Reveals Extensive Remodeling of mRNA Isoforms and Regulatory Networks

****Journal:**** PLOS ONE

Dear Dr. Coller and Reviewers,

We sincerely thank you for your thoughtful and constructive comments on our manuscript. These insights have been invaluable in strengthening our work. We have carefully addressed each point raised and provided a point-by-point response below. All changes in the revised manuscript are marked with track changes; newly added content is highlighted in blue font for clarity.

### ****Response to Reviewer #1****

| **Reviewer Comment** | **Author Response** | **Manuscript Changes** |
| --- | --- | --- |
| ****1. Lines 91-92: Please cite the reference for the CSE preparation method.**** | We thank the reviewer for this suggestion. We have added a citation to a well-established method for CSE preparation. | Added reference [20] (Sampilvanjil et al., 2020) at the end of the first sentence in Section 2.1. |
| ****2. Lines 92-95: Please provide the rationale for using the "Huanghelou" cigarette brand and discuss whether its specific composition is representative.**** | We appreciate this important point. Huanghelou is a commercially available Chinese cigarette brand with tar (10 mg) and nicotine (0.9 mg) content typical of standard research cigarettes used in similar studies. We have added a brief justification and acknowledged that different brands may have compositional variations, which we note as a limitation. | Added to Section 2.1: "This brand was selected as a representative commercially available cigarette with tar and nicotine content (10 mg and 0.9 mg per cigarette, respectively) comparable to standard research cigarettes used in previous studies [20, 21]. We acknowledge that different cigarette brands may have compositional variations, which represents a limitation of this study." |
| ****3. Lines 106-111: Please specify the source of the cell line and include an ethical statement.**** | We thank the reviewer for highlighting this omission. We have now clearly stated the cell line source (ATCC) and added an ethical statement confirming that no human or animal subjects were involved. | Added to Section 2.2: " The immortalized human aortic smooth muscle cell line (HASMC, CP-H081Y) was purchased from Wuhan Procell Biotechnology Co., Ltd‌. The cell line was authenticated by the supplier and tested negative for mycoplasma contamination. As this study used a commercially available cell line with no human or animal subjects, ethical approval was not required." |
| ****4. Lines 124-146: Please provide additional methodological details regarding RNA extraction and ONT library preparation/sequencing.**** | We apologize for the insufficient clarity and organization of the original version, which lacked detailed RNA preparation steps. We have expanded and revised this section accordingly. | Added to Section 2.3: Revised Total RNA was extracted from～1x10^5 cells using total RNA extraction Kit (DP431, TIANGEN, China), resulting 1.6-1.8 μg total RNA for each sample.  We added “ONT library preparation steps with specific reagent volumes (9 μL RNA, 1 μL VNP primer, etc.) and barcoding kit (SQK-NBD114.96).” as the starting sentence for **library preparation.**  **Sequencing part was separated as and independent paragraph：“Each barcoded DNA was purified using 1× Ampure XP Beads, eluted in 20 μl of nuclease free water and quantified using Qubit. A total of 300 fmol of the adapter-ligated library was loaded onto a PromethION flow cell (vR10.4.4) and sequenced on a PromethION 2 Solo platform (Oxford Nanopore Technologies, Oxford, UK).”** |
| ****5. In Figure 1B, it is recommended to annotate the specific p-values.**** | We have updated Figure 1B to include exact p-values for each comparison. | Revised Figure 1B and legend：the exact p-values for each comparison was listed at the upper-right corner. |
| ****6. There is some inconsistency between the description in the main text and the data presented in Figure 1. Please verify and ensure consistency.**** | We have carefully reviewed all figure descriptions and confirmed consistency. One minor discrepancy was found (number of NIC isoforms) and corrected. | Corrected text in Results Section 3.1: Corrected the previously wrong figure citation (Figure 1C-1G). We added the description “CSE treatment led to slightly more novel isoforms (Figure 1D)” |
| ****7. In the Discussion, please briefly address the potential functions of the newly identified transcripts in the cellular response to CSE.**** | We have added a paragraph discussing the potential functional roles of novel transcripts based on their domain and pathway enrichment. | Added to Discussion 2^nd^ paragraph: "we identified 11,222 novel transcripts that substantially expand the known transcriptome. Functional annotation suggests their involvement in diverse cellular processes: enrichment in metabolic pathways implicates them in CSE-induced metabolic reprogramming, while their presence among ribosomal and spliceosomal components points to roles in protein synthesis and RNA processing. Notably, these novel transcripts were enriched in pathways associated with Huntington's, Parkinson's, and Alzheimer's diseases, suggesting shared molecular mechanisms—such as mitochondrial dysfunction, oxidative stress, and impaired autophagy—between smoking-induced vascular pathology and neurodegeneration. Furthermore, Pfam domain analysis revealed that many novel isoforms contain zinc finger or RRM domains, indicating potential RNA-binding activity and possible involvement in post-transcriptional regulatory networks. " |
| ****8. Some statements in the text are overly definitive. A more nuanced phrasing is recommended.**** | We agree and have softened definitive language throughout the manuscript. | Replaced "definitive" with "comprehensive" in title and throughout; replaced "driven by" with "associated with" or "accompanied by"; replaced "establishes" with "suggests" or "indicates" in conclusions. |
| ****9. Please verify all abbreviations used throughout the manuscript.**** | We have conducted a thorough review of all abbreviations and ensured they are defined at first use and used consistently. Inconsistent usage of terminology has been corrected throughout the manuscript. | The abbreviation "HASMCs" is now used consistently when referring to the cells used in this study, whereas "VSMC" is reserved for the broader cell type. Furthermore, plural forms of abbreviations such as "DEGs" and "APAs" have been standardized. The full name of ASE，alternative splicing event, has been added. |

We thank Reviewer #1 for the positive assessment and the constructive suggestions.

### ****Response to Reviewer #2****

We thank Reviewer #2 for the thorough and insightful critique, which has helped us significantly improve the manuscript. We acknowledge that our study is primarily an omics discovery work, and we have taken care to address each concern while maintaining the integrity of our findings.

#### ****1.1 Deficiencies in Experimental Design****

| **Reviewer Comment** | **Author Response** | **Manuscript Changes** |
| --- | --- | --- |
| ****1. Single concentration for sequencing, lacking concentration-gradient validation.**** | We acknowledge this limitation. Our rationale for selecting 0.6% CSE was based on the dose-response curve (Figure 1B), where 0.6% represents the midpoint of the cytotoxic phase. We have now explicitly stated this rationale and acknowledged that concentration-dependent isoform changes warrant future investigation. | Added to Section 2.2: " Based on the dose-response curve of the CSE cytotoxicity (Figure 1B), the midpoint concentration (0.6%) was selected for subsequent transcriptomic analysis. "  We added a limitation paragraph in Discussiod, which includes "First, sequencing was performed at a single CSE concentration (0.6%). While this concentration represents the midpoint of the cytotoxic phase based on our dose-response data, future studies examining concentration-dependent isoform changes across the full range of CSE concentrations will be valuable to fully elucidate the molecular transition from proliferation promotion to cytotoxicity." |
| ****2. Limited cellular model without in vivo or clinical validation.**** | This is an important limitation that we now explicitly acknowledge. As an initial discovery study, our goal was to provide a high-resolution map in a controlled in vitro system. We have added this limitation to the Discussion and suggested future directions for validation. | Added to Discussion (limitation paragraph): " Second, our study used a single immortalized cell line without validation in primary cells, animal models, or clinical samples. While this controlled system enabled comprehensive transcriptomic profiling, the translational relevance of our findings—particularly the novel isoforms and splicing events—requires confirmation in more complex systems such as smoke-exposed mouse aortas or vascular tissues from human smokers.” |
| ****3. Absence of functional validation experiments.**** | We agree that functional validation is essential for establishing causality. We have clarified that the current study is intended as a discovery-phase resource, providing a foundation for targeted functional studies. We have softened causal language throughout. | Removed causal language (e.g., "driven by" → "associated with") throughout Results and Discussion.  Added to Discussion (limitation paragraph): "Third, as a discovery-oriented study, our work does not include functional validation of specific isoforms or splicing events. However, the comprehensive catalog of isoform-level changes we provide—including 269 differential splicing events and 11,222 novel transcripts—offers a rich resource for future hypothesis-driven investigations. Targeted experiments such as isoform-specific knockdown, splice-switching antisense oligonucleotides, or 3'UTR reporter assays will be necessary to establish the functional consequences of the specific events identified here." |

#### ****1.2 Inadequacies in Data Analysis and Interpretation****

| **Reviewer Comment** | **Author Response** | **Manuscript Changes** |
| --- | --- | --- |
| ****1. Superficial characterization of novel transcripts.**** | We acknowledge that our characterization is primarily bioinformatic. We have added a discussion of the limitations and suggested future experimental validation. We have also clarified that database matches (NR/UniProt) provide strong evidence for protein-coding potential. | Added to Discussion (limitation paragraph): " Fourth, while our bioinformatic characterization suggests that the majority of novel transcripts are protein-coding (87.66% with database matches), experimental validation—such as RT-PCR with Sanger sequencing or proteomic confirmation—will be essential to confirm their existence and coding potential. " |
| ****2. Shallow mechanistic interpretation of APA and AS.**** | We agree that mechanistic depth is limited in this discovery study. We have softened interpretations and reframed them as hypotheses for future testing. | Added to Discussion (limitation paragraph):  "Third, as a discovery-oriented study, our work does not include functional validation of specific isoforms or splicing events. However, the comprehensive catalog of isoform-level changes we provide offers a rich resource for future hypothesis-driven investigations. Targeted experiments such as isoform-specific overexpression, splice-switching antisense oligonucleotides, or 3'UTR reporter assays will be necessary to establish the functional consequences of the specific events identified here.”  “...Similarly, the observed 3'UTR remodeling suggests—but does not prove—that miRNA or RBP binding may be altered; mechanistic studies (e.g., luciferase reporter assays, CLIP-seq) are needed to confirm functional consequences.” |
| ****3. Internal inconsistencies and annotation errors.**** | We sincerely apologize for these errors. We have corrected all identified issues. | ****Critical correction****: Changed "human airway smooth muscle cells" to "human aortic smooth muscle cells" in Section 3.4 title and throughout.  ****Gene symbol correction****: "RNP14" → "RNF14" (Figure 5F legend). (No other gene symbol errors were identified upon verification.)  **Consistency between results text and figure legend related to PSI (Section 3.5):** (1) Text change “Comparison of PSI values across splicing types showed that alternative 3′ splice site (A3) events exhibited a relatively larger median PSI difference between conditions compared to other splicing types.” (2) Added to legend (Figure 5). “Black dots indicate individual outlier events. No statistic difference was evident between CSE-exposed and control samples.” |

#### ****1.3 Flaws in Mechanistic Reasoning and Logical Coherence****

| **Reviewer Comment** | **Author Response** | **Manuscript Changes** |
| --- | --- | --- |
| ****1. Confusing correlation with causation.**** | We have systematically replaced causal language with correlational/associational language throughout the manuscript. | Global changes: "driven by" → "associated with"; "executed through" → "accompanied by"; "establishes" → "indicates" or "suggests". Added clarifying statement at the end of Results: "It should be noted that these findings represent correlative associations; causal relationships require functional validation." |
| ****2.Internal logical contradiction. The study claims that "cells attempt to proliferate and survive" despite significantly reduced viability under high CSE concentrations. Upregulation of cell-cycle genes does not equate to active proliferation; a more biologically plausible interpretation is dysregulated cell-cycle checkpoints and mitotic failure under stress.**** | We thank the reviewer for this insightful observation. We fully agree that upregulation of cell-cycle genes does not indicate active proliferation, and our original phrasing was indeed misleading. We have revised the Discussion accordingly, removing the teleological interpretation and replacing it with a more accurate description based on the data. | Revised Discussion: "The upregulation of ferroptosis and cell division genes, coupled with the simultaneous suppression of oxidative phosphorylation, glutathione metabolism, and antiviral defense pathways, reveals a complex transcriptional response to CSE exposure (Figure 2). The increased expression of cell-cycle genes, however, does not reflect active proliferation given the observed reduction in cell viability. Rather, it likely indicates dysregulated cell-cycle checkpoints and mitotic stress—a state in which cell-cycle progression is aberrantly initiated but cannot be successfully completed. This interpretation is consistent with the enrichment of genes involved in chromosome segregation and mitotic regulation among the differentially expressed transcripts (Data not shown). Concurrently, the suppression of oxidative phosphorylation and glutathione metabolism points to a fundamental collapse in energy production and antioxidant defense. Together, these perturbations converge on the activation of specific cell death pathways, including ferroptosis." |
| ****3. Weak regulatory network analysis.**** | We thank the reviewer for this critical comment. We have constructed a core regulatory network for five transcription factors CREM, HBP1, ZNF273, ZNF595, ZNF684 identified in figure 6C (both DET and AS regulated) . We constructed the coexpression network between them and all DEGs, and also identified the target genes of CREM and HBP1 from database. The results support our hypothesis of a feed-forward cascade driven by post-transcriptional regulation . | We have added a new Section 3.7: "Core Regulatory Network of Transcription Factors Associated with Differential Expression and Alternative Splicing " (page ) |

#### ****1.4 Writing and Academic Formatting Issues****

We thank Reviewer #2 for the meticulous language review. We have addressed each specific point and conducted a comprehensive language edit of the entire manuscript.

| **Reviewer Comment** | **Author Response** | **Manuscript Changes** |
| --- | --- | --- |
| ****Line 15: "demonstrates" → "exerts"**** | Corrected. | Abstract, Line 1: "Cigarette smoke extract (CSE) ****exerts**** paradoxical concentration-dependent effects..." |
| ****Line 19: "Following CSE treatment (0.2%-0.8%)" → "after treatment with CSE at concentrations ranging from 0.2% to 0.8%"**** | Revised for clarity. | Abstract, Lines 3-4: We removed the concentration range from this sentence because we only performed ONT sequencing at one CSE concentration. |
| ****Line 19: "We confirmed low-concentration CSE..." → "We confirmed that low-concentration CSE..."**** | Added "that". | Abstract, Line 4: "We confirmed ****that**** low-concentration CSE..." |
| ****Line 20: "slightly promoted viability" → "slightly promoted cell viability"**** | Added "cell". | Abstract, Line 5: "...slightly promoted ****cell**** viability..." |
| ****"vascular damage" → "vascular injury"**** | Changed for consistency. | Abstract, Last line: "...cigarette smoke-mediated vascular ****injury****." |

****Introduction Section Corrections:****

| **Reviewer Comment** | **Author Response** | **Manuscript Changes** |
| --- | --- | --- |
| ****1. "are extensively documented" → "have been extensively documented"**** | Corrected. | Introduction, Line 2: "...have been extensively documented..." |
| ****2. "a subset persists" → "a subset of genes persists"**** | Corrected. | Introduction, Line 4: "...a ****subset of genes**** persists in an altered state..." |
| ****3. "isofrom" → "isoform"**** | Corrected spelling. | Introduction, Line 8: "...which lacks full-length ****isoform**** information." |
| ****4. "data... which lacks" → "data... which lack"**** | Subject-verb agreement corrected. | Introduction, Line 9: "...short-read sequencing data, ****which lack**** full-length isoform information." |
| ****5. "RNA binding protein" → "RNA-binding proteins"**** | Hyphen added. | Introduction, Line 10: "...RNA-binding proteins..." |
| ****6. "in first and last exons" → "in the first and last exons"**** | Added definite article. | Introduction, Line 11: "...in ****the**** first and last exons..." |
| ****7. "Vascular Smooth Muscle Cells (VSMCs)" → "Vascular smooth muscle cells (VSMCs)"**** | Corrected capitalization. | Introduction, “Vascular smooth muscle cells (VSMCs)...” |
| ****8. "is deeply implicated" → "is critically involved"**** | Improved collocation. | Introduction, Line 12: "...is critically involved." |
| ****9. "human aortic smooth muscle cells (HASMC)" → "human aortic smooth muscle cells (HASMCs)"**** | Pluralized abbreviation. | ****Changed to “human aortic smooth muscle cells (HASMCs)"**** |
| ****10. "Our study yields" → "Our study yielded"**** | Tense consistency. | Introduction, Line 13: "Our study ****yielded**** a high-resolution map..." |
| ****11. "differential expressed transcript isoforms" → "differentially expressed transcript isoforms"**** | Corrected adverb form. | Throughout: Verified all instances of "differentially expressed." |

****Additional Language Edits:****

We have conducted a comprehensive language review of the entire manuscript with the help of a professional scientific editing service. Key corrections include:

- Standardized all gene symbols to official HGNC nomenclature
- Verified consistent abbreviation usage throughout
- Corrected subject-verb agreement errors (e.g., "data" as plural)
- Improved sentence flow and eliminated redundancy
- Added missing articles (a, an, the) where needed
- Ensured consistent tense usage (past tense for methods/results, present for established knowledge)

### ****Response to Journal Requirements****

| **Requirement** | **Author Response** |
| --- | --- |
| ****1. PLOS ONE style requirements**** | We have formatted the manuscript according to the PLOS ONE style templates, including file naming conventions. |
| ****2. Code sharing guidelines**** | Most analyses were performed using publicly available software with standard parameters as described in the Methods section; custom code will be provided upon request. |
| ****3. Financial disclosure clarification**** | We confirm that the funder (The General Hospital of Ningxia Medical University, Grant No. 2024AAC03657) provided support in the form of salaries for author Wei Gou, but did not have any additional role in the study design, data collection and analysis, decision to publish, or preparation of the manuscript. The specific roles of the authors are articulated in the 'Author Contributions' list that we submitted originally. We request that the online submission form be updated accordingly. |
| ****4. Title consistency**** | The title has been verified to match between the online submission form and the manuscript file. |
| ****5. Data availability**** | The raw sequencing data generated in this study have been deposited in the NCBI Sequence Read Archive (SRA) under BioProject accession number PRJNA1440235. |

We believe that these revisions have substantially strengthened the manuscript and addressed all concerns raised by the reviewers. We are grateful for the opportunity to improve our work and hope that it is now suitable for publication in PLOS ONE.

Sincerely,

ZhiPeng Hu (on behalf of all authors)
