# Supplementary material for: A comprehensive full-length transcriptome landscape of cigarette smoke-exposed HASMCs reveals extensive remodeling of mRNA isoforms and regulatory networks
Source: PLoS One. 2026 Sep 21;21(9):e0354604. doi: 10.1371/journal.pone.0354604 (PMC13592704; doi:10.1371/journal.pone.0354604)
Supplement: Supplementary file 4 [file pone.0354604.s010.docx]

The scientific validity of the conclusion is insufficient; it is recommended to further refine the scientific conclusions.

Keywords are imprecise.
