# Supplementary material for: A comprehensive full-length transcriptome landscape of cigarette smoke-exposed HASMCs reveals extensive remodeling of mRNA isoforms and regulatory networks
Source: PLoS One. 2026 Sep 21;21(9):e0354604. doi: 10.1371/journal.pone.0354604 (PMC13592704; doi:10.1371/journal.pone.0354604)
Supplement: Peer review history

# Peer review: Decision Letter 0

**DOI:** 10.1371/journal.pone.0354604.r001

**Authors:** Hilary A. Coller

6 Feb 2026

Dear Dr. Hu,

Thank you for submitting your manuscript to PLOS ONE. After careful consideration, we feel that it has merit but does not fully meet PLOS ONE’s publication criteria as it currently stands. Therefore, we invite you to submit a revised version of the manuscript that addresses the points raised during the review process.

Please submit your revised manuscript by Mar 23 2026 11:59PM. If you will need more time than this to complete your revisions, please reply to this message or contact the journal office at plosone@plos.org. When you're ready to submit your revision, log on to https://www.editorialmanager.com/pone/ and select the 'Submissions Needing Revision' folder to locate your manuscript file.

- A letter that responds to each point raised by the academic editor and reviewer(s). You should upload this letter as a separate file labeled 'Response to Reviewers'.
- A marked-up copy of your manuscript that highlights changes made to the original version. You should upload this as a separate file labeled 'Revised Manuscript with Track Changes'.
- An unmarked version of your revised paper without tracked changes. You should upload this as a separate file labeled 'Manuscript'.

If you would like to make changes to your financial disclosure, please include your updated statement in your cover letter. Guidelines for resubmitting your figure files are available below the reviewer comments at the end of this letter.

If applicable, we recommend that you deposit your laboratory protocols in protocols.io to enhance the reproducibility of your results. Protocols.io assigns your protocol its own identifier (DOI) so that it can be cited independently in the future. For instructions see: https://journals.plos.org/plosone/s/submission-guidelines#loc-laboratory-protocols. Additionally, PLOS ONE offers an option for publishing peer-reviewed Lab Protocol articles, which describe protocols hosted on protocols.io. Read more information on sharing protocols at https://plos.org/protocols?utm_medium=editorial-email&utm_source=authorletters&utm_campaign=protocols.

We look forward to receiving your revised manuscript.

Kind regards,

Hilary A. Coller

Academic Editor

PLOS One

Journal Requirements:

When submitting your revision, we need you to address these additional requirements.

1. Please ensure that your manuscript meets PLOS ONE's style requirements, including those for file naming. The PLOS ONE style templates can be found at

https://journals.plos.org/plosone/s/file?id=wjVg/PLOSOne_formatting_sample_main_body.pdf and

https://journals.plos.org/plosone/s/file?id=ba62/PLOSOne_formatting_sample_title_authors_affiliations.pdf

2. Please note that PLOS One has specific guidelines on code sharing for submissions in which author-generated code underpins the findings in the manuscript. In these cases, we expect all author-generated code to be made available without restrictions upon publication of the work. Please review our guidelines at https://journals.plos.org/plosone/s/materials-and-software-sharing#loc-sharing-code and ensure that your code is shared in a way that follows best practice and facilitates reproducibility and reuse.

3. Thank you for stating the following financial disclosure:

“The General Hospital of Ningxia Medical University(No.2024AAC03657), Wei Gou. The funders had no role in study design, data collection and analysis, decision to publish, or preparation of the manuscript.”

We note that one or more of the authors is affiliated with the funding organization, indicating the funder may have had some role in the design, data collection, analysis or preparation of your manuscript for publication; in other words, the funder played an indirect role through the participation of the co-authors. If the funding organization did not play a role in the study design, data collection and analysis, decision to publish, or preparation of the manuscript and only provided financial support in the form of authors' salaries and/or research materials, please do the following:

a. Review your statements relating to the author contributions, and ensure you have specifically and accurately indicated the role(s) that these authors had in your study. These amendments should be made in the online form.

b. Confirm in your cover letter that you agree with the following statement, and we will change the online submission form on your behalf:

“The funder provided support in the form of salaries for authors [insert relevant initials], but did not have any additional role in the study design, data collection and analysis, decision to publish, or preparation of the manuscript. The specific roles of these authors are articulated in the ‘author contributions’ section.

4. Please amend either the title on the online submission form (via Edit Submission) or the title in the manuscript so that they are identical.

5. If the reviewer comments include a recommendation to cite specific previously published works, please review and evaluate these publications to determine whether they are relevant and should be cited. There is no requirement to cite these works unless the editor has indicated otherwise.

Additional Editor Comments:

Please address the reviewers' comments.

[Note: HTML markup is below. Please do not edit.]

Reviewers' comments:

Reviewer's Responses to Questions

**Comments to the Author**

1. Is the manuscript technically sound, and do the data support the conclusions?

Reviewer #1: Yes

Reviewer #2: Yes

**********

2. Has the statistical analysis been performed appropriately and rigorously?

Reviewer #1: Yes

Reviewer #2: Yes

**********

3. Have the authors made all data underlying the findings in their manuscript fully available?

The PLOS Data policy

Reviewer #1: No

Reviewer #2: No

**********

4. Is the manuscript presented in an intelligible fashion and written in standard English?

Reviewer #1: Yes

Reviewer #2: Yes

**********

Reviewer #1: This study employs Oxford Nanopore long-read sequencing to systematically analyze the impact of cigarette smoke extract (CSE) on the full-length transcriptome of human aortic smooth muscle cells (HASMCs), revealing CSE-induced transcript isoform remodeling, alternative polyadenylation (APA), and alternative splicing (AS) events. The research design is sound, the data are comprehensive, and the work demonstrates a degree of novelty. However, several areas require refinement, including methodological descriptions, ethical statements, figure correspondence, and discussion depth.

1. Lines 91-92: Please cite the reference for the CSE preparation method.

2. Lines 92-95: Please provide the rationale for using the "Huanghelou" cigarette brand and discuss whether its specific composition is representative.

3. Lines 106-111: Please specify the source of the cell line and include an ethical statement.

4. Lines 124-146: Please provide additional methodological details regarding RNA extraction and ONT library preparation/sequencing.

5. In Figure 1B, it is recommended to annotate the specific p-values.

6. There is some inconsistency between the description in the main text and the data presented in Figure 1. Please verify and ensure consistency.

7. In the Discussion, please briefly address the potential functions of the newly identified transcripts in the cellular response to CSE.

8. Some statements in the text are overly definitive. A more nuanced phrasing is recommended.

9. Please verify all abbreviations used throughout the manuscript.

Reviewer #2: This study provides a high-resolution, isoform-resolved transcriptomic landscape for smoking-induced vascular injury, with clear and valuable innovations. However, its conclusions are limited by weak causality and incomplete validation.

1. Major Limitations

This is essentially an omics-screening study, and it exhibits common weaknesses in such research, including an incomplete evidence chain, logical discontinuities, and experimental design constraints. Some technical and analytical flaws further compromise the reliability of the conclusions.

1.1 Deficiencies in Experimental Design

1.Single concentration for sequencing, lacking concentration-gradient validation. Although the study confirms the biphasic effects of CSE, long-read sequencing was performed only at 0.6% CSE. It remains unclear whether isoform changes occur in a concentration-dependent manner, and the mechanistic differences between low-concentration proliferation promotion and high-concentration cytotoxicity are not addressed, disconnecting the core scientific question from the sequencing data.

2.Limited cellular model without in vivo or clinical validation. Only an immortalized HASMC cell line was used. Key isoforms, APA and AS events were not validated in primary HASMCs, animal models (e.g., aortas from smoke-exposed mice), or clinical samples (vascular tissues or peripheral blood from smoking patients), limiting translational relevance.

3.Absence of functional validation experiments. All conclusions remain at the level of observational omics analysis. No isoform-specific gain- or loss-of-function assays, splice-site editing, or 3'UTR truncation/extension experiments were performed to establish causality between post-transcriptional dysregulation and CSE-induced cytotoxicity. Only correlative evidence is provided.

1.2 Inadequacies in Data Analysis and Interpretation

1.Superficial characterization of novel transcripts. The study only reports exon numbers, database alignment rates, and pathway enrichment for novel transcripts, without experimental validation (e.g., RT-PCR for full-length sequences) or functional comparison with known isoforms. The biological significance of novel transcripts remains poorly defined.

2.Shallow mechanistic interpretation of APA and AS. The study merely lists differential events and enriched pathways, without exploring the underlying molecular mechanisms: whether 3'UTR lengthening or shortening affects miRNA/RBP binding and mRNA stability; whether AS dysregulation of transcription factors alters nuclear localization or target gene specificity; or whether APA and AS function coordinately.

3.Internal inconsistencies and annotation errors. In Section 3.4, human aortic smooth muscle cells were incorrectly labeled as human airway smooth muscle cells. Figure legends are inconsistent with result descriptions, and statistical annotations (e.g., significance of PSI values) are incomplete.

1.3 Flaws in Mechanistic Reasoning and Logical Coherence

1.Confusing correlation with causation. The study concludes that post-transcriptional dysregulation drives CSE-induced cytotoxicity solely based on co-enrichment of ferroptosis genes and post-transcriptional events, without rescue experiments to support causal relationships.

2.Internal logical contradiction. The study claims that “cells attempt to proliferate and survive” despite significantly reduced viability under high CSE concentrations. Upregulation of cell-cycle genes does not equate to active proliferation; a more biologically plausible interpretation is dysregulated cell-cycle checkpoints and mitotic failure under stress.

3.Weak regulatory network analysis. Overlap among DETs, DEGs, and AS genes is presented only via Venn diagrams, without construction of a core regulatory network. The hypothesis of a feed-forward cascade driven by post-transcriptional regulation lacks supportive evidence.

1.4 Writing and Academic Formatting Issues

Frequent grammatical and typographical errors, inconsistent gene symbols (e.g., ACTRA1 instead of ACTA1, RNP14 instead of RNF14), inconsistent cell line abbreviations, and mismatched figure legends reduce academic rigor. The discussion overstates conclusions by claiming a “fundamental shift” in understanding without sufficient functional and in vivo evidence.

Line 15:Cigarette smoke extract (CSE) demonstrates paradoxical concentration-dependent effects.....

"Demonstrates" is often used for people or research, which is not suitable for a substance. Therefore, it should be replaced with "exerts".

Line 19：Following CSE treatment (0.2%-0.8%)

Change to: after treatment with CSE at concentrations ranging from 0.2% to 0.8%.

Line 19：We confirmed low-concentration CSE...

It should be changed to: We confirmed that low-concentration CSE......

Line 20: Original sentence: slightly promoted viability → revised to slightly promoted cell viability

Original sentence: slightly promoted viability → revised to slightly promoted cell viability.

Original sentence:vascular damage → vascular injury

In the Introduction section.

1. The pathophysiological consequences of smoking are extensively documented......

It should be changed to:......have been extensively documented......

2.Reference ambiguity (semantic inconsistency) a subset persists in an altered state. The absence of a central noun in "subset" makes it unclear to readers whether it refers to genes, transcripts, or proteins. It should be revised to "a subset of genes.".

3.Spelling mistake (fatal flaw) - "isofrom" should be spelled as "isoform".

4.…the short-read sequencing data, which lacks full-length isoform information.

The antecedent is "data" (a plural concept, treated as plural in academic contexts), and the predicate of the attributive clause should be changed to "which lack".

5.Fixed terminology error: RNA binding protein → RNA-binding proteins (with hyphen for standardization).

6.Original sentence: ......particularly in first and last exons......

The ordinal number must be preceded by a definite article: the first and last exons.

7.1. Inconsistent use of uppercase and lowercase letters

For example: Vascular Smooth Muscle Cells (VSMCs) .

Unless at the beginning of a sentence, the names of specialized cells do not need to be written in all capital letters. The standard writing is: Vascular smooth muscle cells (VSMCs).

8.The collocation is awkward. For example, the phrase "a process in which post-transcriptional regulation is deeply implicated"

Fixed collocation: "implicated in". The semantic meaning and collocation here are not natural. It is more accurate to change it to "is critically involved".

9.Error in noun singular and plural. For example, the term "human aortic smooth muscle cells (HASMC)". Cells are countable in the plural, so the abbreviation should be unified as: HASMCs.

10.Inconsistent tense.

The previous text described the experimental operation in the past tense, but here it suddenly switches to the present tense:Our study yields a high-resolution map… Should be changed to past tense: Our study yielded.

11.Misuse of words. The standard academic term for "differentially expressed transcript isoforms" is "differentially expressed transcript isoforms".

There are also many such errors in the Material and Methods, Results, and Discussion sections. I hope the author can carefully revise and correct them

**********

what does this mean?). If published, this will include your full peer review and any attached files.

If you choose “no”, your identity will remain anonymous but your review may still be made public.

**Do you want your identity to be public for this peer review?**  For information about this choice, including consent withdrawal, please see our Privacy Policy

Reviewer #1: No

Reviewer #2: No

**********

[NOTE: If reviewer comments were submitted as an attachment file, they will be attached to this email and accessible via the submission site. Please log into your account, locate the manuscript record, and check for the action link "View Attachments". If this link does not appear, there are no attachment files.]

To ensure your figures meet our technical requirements, please review our figure guidelines: https://journals.plos.org/plosone/s/figures

You may also use PLOS’s free figure tool, NAAS, to help you prepare publication quality figures: https://journals.plos.org/plosone/s/figures#loc-tools-for-figure-preparation.

NAAS will assess whether your figures meet our technical requirements by comparing each figure against our figure specifications.

Attachment Submitted filename: Review-plos one.docx

# Author comment: Author response to Decision Letter 1

**DOI:** 10.1371/journal.pone.0354604.r002

24 Mar 2026

The letter that responds to each point raised by the academic editor and reviewers has been uploaded.

Attachment Submitted filename: ONT-responses-R1-final.docx

# Peer review: Decision Letter 1

**DOI:** 10.1371/journal.pone.0354604.r003

**Authors:** Hilary A. Coller

9 Jul 2026

A Comprehensive Full-Length Transcriptome Landscape of Cigarette Smoke-Exposed HASMCs Reveals Extensive Remodeling of mRNA Isoforms and Regulatory Networks

PONE-D-25-67222R1

Dear Dr. Hu,

We’re pleased to inform you that your manuscript has been judged scientifically suitable for publication and will be formally accepted for publication once it meets all outstanding technical requirements.

Within one week, you’ll receive an e-mail detailing the required amendments. When these have been addressed, you’ll receive a formal acceptance letter and your manuscript will be scheduled for publication.

An invoice will be generated when your article is formally accepted. Please note, if your institution has a publishing partnership with PLOS and your article meets the relevant criteria, all or part of your publication costs will be covered. Please make sure your user information is up-to-date by logging into Editorial Manager at Editorial Manager® and clicking the ‘Update My Information' link at the top of the page. For questions related to billing, please contact billing support.

If your institution or institutions have a press office, please notify them about your upcoming paper to help maximize its impact. If they’ll be preparing press materials, please inform our press team as soon as possible -- no later than 48 hours after receiving the formal acceptance. Your manuscript will remain under strict press embargo until 2 pm Eastern Time on the date of publication. For more information, please contact onepress@plos.org.

Kind regards,

Hilary A. Coller

Academic Editor

PLOS One

Additional Editor Comments (optional):

Thank you for submitting a revised manuscript, which we have decided to accept. We recognize that not all of the reviewers were supportive of publication. A review of similar manuscripts published in PLOS One revealed that when we applied consistent standards across manuscripts, the manuscript should be accepted. Thank you for submitting to PLoS One.

Reviewers' comments:

Reviewer's Responses to Questions

**Comments to the Author**

Reviewer #1: All comments have been addressed

**********

2. Is the manuscript technically sound, and do the data support the conclusions?

Reviewer #1: Yes

**********

3. Has the statistical analysis been performed appropriately and rigorously?

Reviewer #1: Yes

**********

4. Have the authors made all data underlying the findings in their manuscript fully available?

The PLOS Data policy

Reviewer #1: Yes

**********

5. Is the manuscript presented in an intelligible fashion and written in standard English?

Reviewer #1: No

**********

Reviewer #1: (No Response)

**********

what does this mean?). If published, this will include your full peer review and any attached files.

If you choose “no”, your identity will remain anonymous but your review may still be made public.

**Do you want your identity to be public for this peer review?**  For information about this choice, including consent withdrawal, please see our Privacy Policy

Reviewer #1: No

**********

Attachment Submitted filename: Review comments-C.docx

# Editor report: Acceptance letter

**DOI:** 10.1371/journal.pone.0354604.r004

**Authors:** Hilary A. Coller

PONE-D-25-67222R1

PLOS One

Dear Dr. Hu,

I'm pleased to inform you that your manuscript has been deemed suitable for publication in PLOS One. Congratulations! Your manuscript is now being handed over to our production team.

At this stage, our production department will prepare your paper for publication. This includes ensuring the following:

* All references, tables, and figures are properly cited

* All relevant supporting information is included in the manuscript submission,

* There are no issues that prevent the paper from being properly typeset

You will receive further instructions from the production team, including instructions on how to review your proof when it is ready. Please keep in mind that we are working through a large volume of accepted articles, so please give us a few days to review your paper and let you know the next and final steps.

Lastly, if your institution or institutions have a press office, please let them know about your upcoming paper now to help maximize its impact. If they'll be preparing press materials, please inform our press team within the next 48 hours. Your manuscript will remain under strict press embargo until 2 pm Eastern Time on the date of publication. For more information, please contact onepress@plos.org.

You will receive an invoice from PLOS for your publication fee after your manuscript has reached the completed accept phase. If you receive an email requesting payment before acceptance or for any other service, this may be a phishing scheme. Learn how to identify phishing emails and protect your accounts at https://explore.plos.org/phishing.

If we can help with anything else, please email us at customercare@plos.org.

Thank you for submitting your work to PLOS ONE and supporting open access.

Kind regards,

PLOS ONE Editorial Office Staff

on behalf of

Dr. Hilary A. Coller

Academic Editor

PLOS One
